# Response of the airways and autonomic nervous system to acid perfusion of the esophagus in patients with asthma: a laboratory study

**DOI:** 10.1186/1471-2466-13-33

**Published:** 2013-06-02

**Authors:** D Lakmali Amarasiri, Arunasalam Pathmeswaran, H Janaka de Silva, Channa D Ranasinha

**Affiliations:** 1 Department of Physiology, Faculty of Medicine, University of Kelaniya, P.O. Box 6, Thalagolla Road, Ragama, Sri Lanka; 2 Department of Public Health, Faculty of Medicine, University of Kelaniya, Ragama, Sri Lanka; 3 Department of Medicine, Faculty of Medicine, University of Kelaniya, Ragama, Sri Lanka; 4 Department of Pharmacology, Faculty of Medicine, University of Kelaniya, Ragama, Sri Lanka

**Keywords:** Asthma, Gastro-esophageal reflux disease, Vagal activity, Spirometry, Autonomic function testing

## Abstract

**Background:**

Gastro-esophageal reflux disease (GERD) predisposes to airway disease through a vagally-mediated esophago-bronchial reflex. This study investigates this vagal response to esophageal acid perfusion.

**Methods:**

40 asthmatics with mild stable asthma participated. Each subject underwent spirometry and autonomic function testing (valsalva maneuver, heart rate response to deep breathing and to standing from supine position) four times: a) before intubation, b) after intubation, and then immediately after perfusion with, in random order, c) concentrated lime juice solution (pH 2–3) and d) 0.9% saline. Subjects were blinded to the solution perfused.

**Results:**

Asthmatics were of mean (SD) age 34.3 years (1.3), and 67.5% of them were females. pH monitoring demonstrated that 20 subjects had abnormal reflux and 20 did not. In each group 10 subjects had a positive GERD symptom score. Following perfusion with acid compared to saline, all subjects showed significant decreases in FEV_1_ and PEFR and significant increases in the mean valsalva ratio and heart rate difference on deep breathing from baseline values, but no changes in FVC or heart rate ratio on standing. There were no significant differences in any of the parameters between subjects with and without reflux.

**Conclusions:**

Acid stimulation of the distal esophagus results in increased parasympathetic activity and concomitant broncho-constriction in asthmatics irrespective of their reflux state. This strengthens the hypothesis that GER triggers asthma-like symptoms through a vagally mediated esophago-bronchial reflex and encourages a possible role for anti-cholinergic drugs in the treatment of reflux-associated asthma.

## Background

Gastro-esophageal reflux disease (GERD) precipitates airway hyper-responsiveness, lung function decrease and asthma-like symptoms. The foregut and respiratory tract have a common embryological origin [1]. It is possible that inflammation in either leads to reflexes that manifest clinically as asthma or GERD [2]. The pathway for triggering ‘asthma-like’ symptoms is the vagal sensory nerves innervating the airways and lungs [3]. The proposed mechanisms are micro-aspiration of acid into airways or an acid induced-esophago-bronchial reflex [4].

Microaspiration of refluxate damages epithelial cells causing an inflammatory response in the airways of patients with GERD. Independent of aspiration, esophageal acid reflux may cause an increase in vagal tone with stimulation of vagal reflexes. Evidence for this vagal-mediated reflex comes from acid perfusion studies [5]. These are performed to simulate the distal esophageal response to acid, without allowing the acid to reflux proximally thereby preventing proximal aspiration. Gastro-esophageal reflux (GER) has been variously reported to have an important effect [6,7], a small effect [8-10] and no effect on pulmonary function [11-13]. Regionally, two studies have reported an increase in airway resistance [14] and decrease in forced expiratory volume in the first second (FEV_1_) [15] following esophageal acid perfusion. Several other studies have also demonstrated bronchial reactivity [16-19]. In two other studies in asthmatics with GER, intra-esophageal acid infusion caused a decrease in peak expiratory flow rate (PEFR) and a drink of dilute hydrochloric acid (HCl) significantly increased airway sensitivity to inhaled histamine in childhood asthma, and this was abolished with pre-treatment with atropine [20].

This suggested a vagal reflex as the most likely explanation for increase in airway hyper-responsiveness and shows that the vagus nerve is involved in the direct vagally mediated reflex, the heightened bronchial reactivity mechanism and the microaspiration theory [1]. Asthmatics with GERD have been shown to have a hypervagal response [21].

The aim of our study was to study the vagal response and pulmonary function in asthmatics following esophageal acid perfusion.

## Methods

The subjects were consecutive asthmatics (American Thoracic Society criteria) [22] recruited from medical clinics of the Colombo North Teaching Hospital, Ragama, Sri Lanka, aged between 18–60 years, who were did not smoke or abuse alcohol and were not diabetic. Additional exclusion criteria were the presence of known esophageal diseases, a history of previous upper gastro-intestinal or pulmonary surgery, or presence of cardiovascular disease. All subjects gave informed written consent before the study.

None of the subjects had had an exacerbation of asthma in the two weeks preceding the study. They were not on any drugs altering heart rate or blood pressure, or if they were these were withheld for 24 hours prior to testing. Oral asthma drugs were withheld for 24 hours and inhaled drugs for 8 hours prior to the study, allowing inhaled or nebulized beta-2 agonists on an as-required basis. Acid suppressing medications were discontinued 7 days and prokinetic agents 48 hours before the study. Antacids were stopped at midnight the day before.

All subjects underwent baseline spirometry, stationary esophageal manometry and 24- hour ambulatory pH monitoring assessment. Spirometry was performed using a portable hand-held spirometer (Micro Plus spirometer, Micro Medical Limited, Rochester, UK). Forced vital capacity (FVC), FEV_1_, and PEFR were recorded after each forced expiratory effort. Stationary esophageal manometry was performed using a water perfused system (Synetics PC Polygraf system, Stockholm, Sweden) according to standard methodology to determine the position of the lower esophageal sphincter (LES). 24-hour esophageal pH monitoring was performed using a dual sensor monocrystalline antimony catheter (Synetics Medical AB, Stolkholm, Sweden), according to standard methodology. The distal sensor of the pH catheter was positioned 5 cm above the superior border of the manometrically determined LES and the proximal sensor 15 cm above the distal sensor. The presence of reflux was assessed according to methodology of DeMeester and Johnson [23] and proximal and distal reflux was determined according to cut-off values derived from a population of healthy controls (Table 1) [24].

**Table 1:** Acid exposure in the esophagus in healthy volunteers

	***Proximal sensor***	**( *n *= *16 *)**	***Distal sensor***	**( *n *= *26 *)**
**Parameter**	**Median (range)**	**95th percentile**	**Median (range)**	**95th percentile**
Total % time pH<4	0.07 (0-0.4)	0.4	0.4 (0.08-1.5)	1.5
% time pH<4 in upright position	0.02 (0-0.4)	0.4	0.2 (0-3.0)	3.0
% time pH<4 in supine position	0.12 (0-0.5)	0.5	0.4 (0.07-2.8)	2.8
Total no. of reflux episodes	6.0 (0-25.0)	25.0	18.0 (2.7-94.2)	94.2
No. of episodes ≥ 5 min	0 (0-1)	1.0	0 (0-2)	2.0
Duration of longest reflux, min	0.9 (0-7.8)	7.8	1.9 (0.5-20.6)	20.6
DeMeester score	0.95 (0.2-4.8)	4.8	3.35 (0.7-11.5)	11.5

The subjects were screened by a previously validated GERD screening score assessing frequency and severity of 7 common upper gastrointestinal symptoms on a 5-point Likert scale. Symptoms included were 1) heartburn 2) regurgitation 3) upper abdominal or chest pain 4) abdominal distension 5) dysphagia 6) cough and 7) belching. A GERD score was calculated as the sum of the products of frequency and severity of each symptom. A cut of score of ≥ 12.5 was considered as a positive GERD score [25].

### Vagal function assessment following esophageal perfusion

The day following removal of the pH catheter, following an overnight fast, a feeding tube (4 mm diameter) was used to deliver acid to the distal esophagus. The tube was inserted to 15 cm above the upper border of the lower esophageal sphincter (LES). Heart rate and ECG were monitored continuously. Blood pressure was measured manually at 15 minute intervals. Asthmatics underwent vagal autonomic function testing and spirometry after intubation (baseline) after a resting period of 30 minutes [21]).

The vagal autonomic function tests [26] performed were:

a) Valsalva maneuver produced by sustaining a forced expiration through a mouthpiece connected to a manometer (40 mmHg) lasting 15 s, following a deep inspiration

b) heart rate variation with quiet and deep breathing (six breaths per minute)

c) heart rate response to standing from supine position

Acid perfusion was performed in a seated position. Each subject underwent esophageal perfusion with a solution of normal saline (0.9% Sodium chloride solution, Baxter, India) or solution of concentrated lime juice (pH 3) alternatively, at a rate of 2 mL/min for 10 minutes [27], using a 50 mL syringe. The patients were not aware of which solution they received. At the end of each infusion period, the patients underwent repeat vagal function testing and spirometry. After each infusion and investigation, a time interval of 1 hour was allowed during which the subjects rested in the seated position.

### Statistical analysis

Demographic characteristics are given as mean ± standard error (SE) and mean (standard deviation). Comparison of data between groups was by unpaired or paired t-test as appropriate. Repeated measures of analysis using a general linear model were used to compare the results of the different continuous variables in the two perfusion periods. Categorical variables were compared using the χ^2^ test or the Fisher Exact test. HR Ratio = Heart rate ratio (30th beat/ 15th beat) on standing from supine position, HR Diff = Heart rate difference between inspiration and expiration during deep breathing at 6 cycles per minute, VR = ratio of maximum heart rate during the valsalva maneuver to the maximum heart rate around the 20th beat after stopping the maneuver. A *P* value of ≤ 0.05 was considered significant. All statistical analysis was performed using SPSS version 10.0 for Windows software (SPSS Inc., Chicago, IL, USA).

This study was approved by the Ethics and Scientific Review Committee of the Faculty of Medicine of the University of Kelaniya, Ragama, Sri Lanka. All procedures were conducted following written informed consent and conform to the Declaration of Helsinki.

## Results

Forty asthmatics were studied; 20 had abnormal reflux on pH monitoring and 20 had no reflux. The two groups of asthmatics with and without reflux were found to be comparable for age, gender and body mass index (Table 2). Of the 20 asthmatics with reflux, 10 scored positive on the GERD symptom score and of the 20 with no demonstrable reflux on pH monitoring, 10 had a positive GERD symptom score. The pH monitoring values of the subjects are given in Table 3.

**Table 2:** Baseline characteristics of subjects

	***Asthmatics without reflux *(*n***=***20*)**	***Asthmatics with reflux *( *n *= *20 *)**	**All asthmatics (n=40)**
Age, yrs	33.6 ± 1.9	34.9 ± 1.9	34.3 ± 1.3
Gender, (M:F)	9:11	6:14	15:25
BMI, (kg/m^2^)	20.6 ± 0.6	20.5 ± 0.7	20.5 ± 0.4
GERD symptom score (frequency x severity)*	22.0 ± 3.2	30.5 ± 4.6	26.2 ± 2.9
DeMeester score*	5.3 ± 0.9	44.2 ± 7.1	24.8 ± 4.7
Asthma severity* (no of subjects)			
Mild intermittent	11	3	14
Mild persistent	6	11	17
Moderate or severe persistent	3	6	9
Asthma medication (no of subjects)	-	-	-
Oral salbutamol	10	13	23
Inhaled salbutamol	6	7	13
Oral theophylline	2	5	7
Oral steroids	2	6	8
Inhaled steroids	6	10	16
Spirometry results, mean ± SE			
FVC (L)	3.3 ± 0.1	3.1 ± 0.1	3.2 ± 0.1
FEV1 (L)	2.7 ± 0.1	2.6 ± 0.1	2.7 ± 0.1
PEFR (L)	4.3 ± 0.2	4.1 ± 0.2	4.2 ± 0.1
Autonomic function testing results		1.1 ± 0.03	1.2 ± 0.02
HR ratio*	1.21 ± 0.04	1.1 ± 0.03	1.2 ± 0.02
HR difference	23.1 ± 1.8	23.1 ± 1.5	23.01 ± 1.2
Valsalva ratio	1.3 ± 0.1	1.2 ± 0.05	1.2 ± 0.04

*BMI* Body Mass Index, GERD gastro-esophageal reflux disease, *FVC* Forced Vital Capacity,* FEV*_1_ Forced Expiratory Volume in 1st second,* PEFR *Peak Expiratory Flow Rate.

*HR Ratio* Heart rate ratio (30th beat/ 15th beat) on standing from supine position.

*HR difference* Heart rate difference between inspiration and expiration during deep breathing at 6 cycles per minute.

*Valsalva ratio* ratio of maximum heart rate during the valsalva maneuver to the maximum heart rate around the 20th beat after stopping the maneuver.

All values mean ± SE unless specified otherwise.

* P <0.05; asthmatics with reflux compared to asthmatics without reflux.

**Table 3:** 24 hour pH monitoring data of subjects

	**SPRP**	**SPRN**	**SNRP**	**SNRN**
**Proximal sensor** (**15cm above LES**)				
Total No of reflux episodes	100.0 ± 23.2	14.7 ± 7.2	87.7 ± 25.3	12.1 ± 2.8
No of reflux episodes> 5min	5.8 ± 1.5	0.6 ± 0.3	3.7 ± 1.2	1.7 ± 0.6
Longest reflux episode	71.5 ± 14.8	13.0 ± 12.8	45.7 ± 17.3	2.9 ± 1.4
Total % of time pH<4	9.8 ± 3.4	0.2 ± 0.05	4.9 ± 1.3	0.3 ± 0.07
% of time pH<4 in upright position	7.0 ± 2.8	0.2 ± 0.06	8.1 ± 2.1	0.3 ± 0.09
% of time pH<4 in supine position	12.1 ± 5.3	0.1 ± 0.0	5.3 ± 2.8	0.3 ± 0.2
Demeester score	45.2 ± 12.1	3.7 ± 2.3	31.1 ± 7.3	2.8 ± 0.4
**Distal sensor** (**5cm above LES**)				
Total No of reflux episodes	103.2 ± 20	31.5 ± 7.3	64.9 ± 13.8	22.7 ± 7.2
No of reflux episodes> 5min	6.4 ± 0.9	10.8 ± 8.1	4.3 ± 0.9	1.6 ± 0.8
Longest reflux episode	58.8 ± 15.2	4.8 ± 1.7	51.2 ± 15.4	2.9 ± 1.1
Total % time pH<4	14.6 ± 4.5	0.7 ± 0.2	8.8 ± 2.0	0.5 ± 0.2
% time pH<4 in upright position	9.7 ± 3.3	1.5 ± 0.7	7.5 ± 2.4	1.1 ± 0.7
% time pH<4 in supine position	20.2 ± 6.2	0.7 ± 0.3	5.9 ± 2.0	0.5 ± 0.2
Demeester score	57.0 ± 12.2	6.3 ± 1.3	31.4 ± 5.4	4.3 ± 1.2

All values as mean ± SE.

*SPRP* GERD symptom positive, reflux positive, *SPRN* GERD symptom positive, reflux negative, *SNRP* GERD symptom negative, reflux positive, *SNRN* GERD symptom negative, reflux negative.

The 40 subjects were randomised: 20 of them received acid perfusion first followed by saline as the second perfusion and the other 20 received the perfusions in reverse order. Comparison of the data at the first and second measurements in individual subjects showed that the order in which the perfusion was given did not affect results.

All asthmatics demonstrated a significant decrease in FEV_1_ and PEFR from the baseline values following perfusion with acid when compared to saline. There was no such change in FVC (Table 4). All asthmatics also showed a significant increase in the mean valsalva ratio and heart rate difference on deep breathing from baseline values following perfusion with acid when compared to saline. There was no change in heart rate ratio on standing (Table 4).

**Table 4:** **Spirometry and vagal function tests**: **baseline and following saline and acid perfusion** (**all asthmatics**; **n**=**40**)

***Parameter***	***Baseline***	***After saline perfusion***	***After acid perfusion***	P value*
				***Saline versus acid perfusion***
**Spirometry** (**L**/**min**)				
FVC	3.2 ± 0.10	3.2 ± 0.10	3.2 ± 0.10	0.084
FEV_1_	2.7 ± 0.08	2.7 ± 0.08	2.6 ± 0.07	0.001
PEFR	4.2 ± 0.14	4.1 ± 0.15	4.0 ± 0.13	0.023
**Vagal function tests**				
Valsalva ratio	1.26 ± 0.04	1.23 ± 0.03	1.31 ± 0.04	0.011
Heart rate ratio on standing from supine position	1.16 ± 0.02	1.14 ± 0.02	1.12 ± 0.02	0.612
Heart rate difference on deep breathing	23.09 ± 1.16	23.6 ± 1.16	26.0 ± 1.40	0.004

* Repeated measures analysis; All values as mean ± SE.

*FVC* Forced Vital Capacity, *FEV*_*1*_ Forced Expiratory Volume in 1st Second, *PEFR* Peak Expiratory Flow Rate.

There was no significant difference in the other vagal or spirometry parameters among asthmatics with and without reflux. The asthmatics with reflux had non-significant trends for higher values in the other vagal parameters and greater decreases in FEV_1_ and PEFR compared to those without reflux (Table 5). Comparing asthmatics with and without positive GERD symptom scores also failed to demonstrate differences in any of the parameters following acid or saline perfusion (Table 5).

**Table 5:** **Pulmonary and vagal function tests of asthmatics** (**categorized according to reflux** (**on pH monitoring**) **and GERD symptom score following distal esophageal acid or saline perfusion**

	***Asthmatics with reflux and a negative GERD symptom score *( *n *= *10 *)**		***Asthmatics with reflux and a positive GERD symptom score *( *n *= *10 *)**		***Asthmatics without reflux and a negative GERD symptom score *( *n *= *10 *)**		**Asthmatics without reflux and a positive GERD symptom score (n=10)**	
	**Saline**	**Acid**	**Saline**	**Acid**	**Saline**	**Acid**	**Saline**	**Acid**
FVC (L)	3.0 ± 0.2	3.0 ± 0.2	3.0 ± 0.2	3.0 ± 0.2	3.4 ± 0.2	3.3 ± 0.2	3.1 ± 0.1	3.1 ± 0.1
FEV_1_ (L)	2.6 ± 0.2	2.4 ± 0.1	2.6 ± 0.2	2.4 ± 0.2	2.8 ± 0.2	2.8 ± 0.1	2.6 ± 0.1	2.6 ± 0.1
PEFR (L/min)	4.1 ± 0.2	4.1 ± 0.2	4.2 ± 0.2	4.1 ± 0.2	4.1 ± 0.3	3.9 ± 0.2	3.8 ± 0.2	3.7 ± 0.2
HR Ratio	1.1 ± 0.02	1.1 ± 0.03	1.1 ± 0.02	1.1 ± 0.03	1.2 ± 0.06	1.2 ± 0.05	1.0 ± 0.02	1.0 ± 0.03
HR diff	23.7 ± 2.3	26.0 ± 2.8	23.7 ± 2.4	26.0 ± 2.8	22.0 ± 2.6	25.2 ± 3.2	24.8 ± 2.8	27.0 ± 3.1
VR	1.2 ± 0.06	1.4 ± 0.1	1.2 ± 0.06	1.4 ± 0.09	1.2 ± 0.1	1.3 ± 0.1	1.3 ± 0.05	1.4 ± 0.09

All values as mean ± SE.

*FVC* Forced Vital Capacity, *FEV*_*1*_ Forced Expiratory Volume in 1st second, *PEFR* Peak Expiratory Flow Rate.

*HR Ratio* Heart rate ratio (30th beat/ 15th beat) on standing from supine position, *HR Diff* Heart rate difference between inspiration and expiration during deep breathing at 6 cycles per minute, *VR* ratio of maximum heart rate during the valsalva maneuver to the maximum heart rate around the 20th beat after stopping the maneuver.

## Discussion

Asthmatics have increased parasympathetic autonomic activity [21,28,29] and GER predisposes to asthma-like airway disease [4]. Studies have demonstrated decrease in pulmonary function [9,14,15] and increase in bronchial hyperresponsiveness [16-19] in asthmatics and showed that cholinergic blockade abolishes this response [20]. A vagally-mediated esophago-bronchial reflex has been suggested [30]. Asthmatics with GER have been shown to have hyper-vagal activity too [21].

This study showed that asthmatics demonstrate a significantly higher vagal and broncho-constrictory response to artificially infused esophageal acid when compared to normal saline. To our knowledge this is the first study where vagal function tests have been performed following esophageal acid infusion in asthmatics.

It also showed that asthmatics with reflux showed a trend for a higher response to the other vagal tests and lower spirometry values in response to esophageal acid perfusion compared to those without reflux. However, the change in values demonstrated after saline and acid perfusion were minute and not significant. Further similar studies are required to assess the clinical significance of these results.

A previous study investigating asthmatics with GER noted that 73%, 31% and 6% demonstrated a hypervagal response during a deep breathing manoeuvre, the Valsalva maneuver, and a tilt test respectively. They suggested that this hypervagal responsiveness may be partially responsible for the airway responses to esophageal acid [21].

The present study demonstrated that FEV_1_ and PEFR significantly decreased after an intra-esophageal infusion of acid compared to saline. In a similar study of patients referred for esophageal complaints, FEV_1_ and the heart rate was seen to significantly decrease after administration of normal saline and acid, however with no statistical differences noted between saline and acid. This response was ablated by cholinergic blockade, supporting the theory of a vagally-mediated esophago-bronchial reflex [20]. Other studies too have reported significant changes in pulmonary function and bronchial hyper-responsiveness following acid perfusion [14,31].

However, several studies have found that pulmonary function does not change following acid perfusion. A similar study investigating 43 patients with bronchial asthma showed that esophageal acid perfusion itself did not cause significant reduction in FEV_1_ but that patients with bronchial hyperreactivity, demonstrated by methacholine challenge showed a decrease. They suggested that the esophago-bronchial reflex studied was not present in patients without bronchial hyperreactivity [32]. Another study investigating 20 asthmatics with reflux reported no change in bronchial responsiveness to bradykinin following acid infusion [33]. A review of 18 studies of GER and acid perfusion in adult asthmatics reported that in asthmatics with GER, the effects of acid perfusion are minimal with only a minority affected. It further stated that asthmatics without symptomatic GER showed no change in spirometry, respiratory resistance or flow-volume indices [5].

It has been suggested that the acid-induced increased bronchial response is only demonstrable in ‘sensitive’ esophagi; that is the ‘damaged’ esophageal mucosa exposes nerve endings that produce an augmented effect [7]. Indeed, previous studies have shown significant increase in airway resistance and decrease in PEF in asthmatics with severe esophagitis [33], but no significant changes in asthmatics without esophagitis [27]. Schan et al. [9] showed that esophageal acid caused a decrease in the PEFR and that esophageal mucosal inflammation assessed by a positive Bernstein test was not required for the airway responses. Though we did not assess endoscopic evidence of reflux in our subjects, we confirmed presence of reflux by two other methods: prolonged ambulatory pH monitoring and a validated GERD symptom score. 24 hour esophageal pH monitoring demonstrates the highest sensitivity [34]. Lack of endoscopic evidence is a limitation in the study. Our data also showed that asthmatics demonstrate a decrease in FEV_1_ following acid perfusion irrespective of reflux status.

We employed the criteria of abnormally increased distal sensor Demeester score as the cut-off to determine reflux positive state. Based on this we had 10 subjects per category. A previous study demonstrated that an increase in the number of reflux events at the level of the distal esophagus is detectable in the reflux induced asthma, while the total score remains normal [35]. Failure to consider this is a limitation of the study.

Previous studies have demonstrated that a more powerful stimulus of concentrated acid (0.1 N) instilled into the esophagus in the presence of esophagitis can induce an immediate increase in respiratory resistance [7] and alteration in respiratory inductance [36]. The present study was able to show a response even with a lower, more physiological concentration of acid. It is unlikely therefore, that the low concentration of acid used in the present study contributed to the lack of significant difference among parameters in asthmatics with and without GER.

Wright *et al*.[20] reported that there was no difference in airway flow in patients with pulmonary disease and in those without respiratory complaints. Hence it was postulated that this cholinergic reflex is universal and present in all individuals. A recent study reporting that esophageal hypersensitivity could be induced and maintained by repeated short duration acid infusion at physiological pH levels (pH 1.8-4) in healthy subjects confirms this statement [37]. Therefore, the response of healthy controls to acid perfusion was not assessed in the present study. The lack of data of healthy subjects and non-asthmatic GERD patients is a limitation.

In the present study, the esophageal catheter was placed at 15 cm above the LES. This is a lower position than in previous studies[19,30,38]. Ideally proximal and distal intra-esophageal pH should have been measured continuously to ensure that esophageal pH was neutral during saline perfusion and low during acid perfusion. We did not insert another probe, as an additional probe would have added to the patients’ discomfort, reduced patient compliance and increased the cost of the study substantially. However esophageal acid clearance time in the asthmatics was 0.08 seconds in the proximal esophagus and 0.14 seconds in the distal esophagus. Therefore we considered that the esophageal pH would have come back to neutral levels during the time period of 2 hours allowed between infusions. In one study investigating airway hyper-responsiveness after acid perfusion, the catheter was placed at a site 15 cm above the LES. Even at this more proximal level of acid perfusion, the acid did not reach the proximal esophagus [19]. Therefore, though we had no facility to monitor the pH of the upper part of the esophagus, it was unlikely that the pharynx was stimulated by acid perfusion and that aspiration of acid had an influence on the results.

None of our study subjects complained of pain during the study (positive Bernstein test). It is possible that infusion of the acid through a tube, bypassing taste input and blinding the individual to the solution may have resulted in this. Other studies have used lower pH values for the acid perfusion study [10,19,25]. We used lime juice at a pH of 2–3 which simulated gastric pH more closely and is more physiological. This could be another reason for the negative Bernstein test. Other studies have also demonstrated a decline in PEFR following acid perfusion that was independent of a positive Bernstein test [9,10].

Pulmonary function testing by spirometry is effort-dependent. Though there was concern that the presence of the tube may have interfered with the results, there was no difference between spirometry parameters before and after intubation.

## Conclusions

Acid stimulation of the distal esophagus results in increased vagal activity and a concomitant broncho-constriction in asthmatics irrespective of their reflux state. This finding further strengthens the hypothesis that GER may trigger asthma-like symptoms through a vagally mediated esophago-bronchial reflex and suggests a role for anti-cholinergic drugs in the treatment of reflux-associated asthma.

## Abbreviations

GERD: Gastro-esophageal reflux disease; FEV1: Forced expired volume in the first second; GER: Gastro-esophageal reflux; PEFR: Peak expiratory flow rate; HCl: Hydrochloric acid; FVC: Forced vital capacity; LES: Lower esophageal sphincter; ECG: Electrocardiography; SE: Standard error; HR Ratio: Heart rate ratio of 30th beat/ 15th beat on standing from supine position; HR Diff: Heart rate difference between inspiration and expiration during deep breathing at 6 cycles per minute; VR: ratio of maximum heart rate during the valsalva maneuver to the maximum heart rate around the 20th beat after stopping the maneuver

## Competing interests

DLA, AP, HJDES and CDR have no conflicts of interest to disclose.

## Authors’ contributions

All authors were involved in study conception, design, manuscript drafting and revision. DLA and AP were involved in statistical analysis. DLA was involved in data acquisition. All authors have given final approval of the version to be published.

## Pre-publication history

The pre-publication history for this paper can be accessed here:

http://www.biomedcentral.com/1471-2466/13/33/prepub

## References

[B1] Harding SM Gastresophageal reflux and asthma: insight into the association J Allergy Clin Immunol 1999 104 2 Pt 1 251 259 10452738 10.1016/s0091-6749(99)70360-x

[B2] Canning BJ Fischer A Neural regulation of airway smooth muscle tone Respir Physiol 2001 125 1–2 113 127 11240156 10.1016/s0034-5687(00)00208-5

[B3] Canning BJ Mazzone SB Reflex mechanisms in gastresophageal reflux disease and asthma Am J Med 2003 115 Suppl 3A 45S 48S 12928074 10.1016/s0002-9343(03)00192-x

[B4] Harding SM Richter JE The role of gastresophageal reflux in chronic cough and asthma Chest 1997 111 5 1389 1402 10.1378/chest.111.5.1389 9149599

[B5] Field SK A critical review of the studies of the effects of simulated or real gastresophageal reflux on pulmonary function in asthmatic adults Chest 1999 115 3 848 856 10.1378/chest.115.3.848 10084501

[B6] Mansfield LE Stein MR Gastresophageal reflux and asthma: a possible reflex mechanism Ann Allergy 1978 41 4 224 226 707849

[B7] Spaulding HS Jr Mansfield LE Stein MR Sellner JC Gremillion DE Further investigation of the association between gastresophageal reflux and bronchoconstriction J Allergy Clin Immunol 1982 69 6 516 521 10.1016/0091-6749(82)90176-2 7076992

[B8] Perpina M Pellicer C Marco V Maldonado J Ponce J The significance of the reflex bronchoconstriction provoked by gastresophageal reflux in bronchial asthma Eur J Respir Dis 1985 66 2 91 97 3972024

[B9] Schan CA Harding SM Haile JM Bradley LA Richter JE Gastresophageal reflux-induced bronchoconstriction An intraesophageal acid infusion study using state-of-the-art technology. Chest 1994 106 3 731 737 10.1378/chest.106.3.731 8082350

[B10] Harding SM Schan CA Guzzo MR Alexander RW Bradley LA Richter JE Gastresophageal reflux-induced bronchoconstriction. Is microaspiration a factor? Chest 1995 108 5 1220 1227 10.1378/chest.108.5.1220 7587420

[B11] Wesseling G Brummer RJ Wouters EF Ten Velde GP Gastric asthma? No change in respiratory impedance during intraesophageal acidification in adult asthmatics Chest 1993 104 6 1733 1736 10.1378/chest.104.6.1733 8252953

[B12] Tan WC Martin RJ Pandey R Ballard RD Effects of spontaneous and simulated gastresophageal reflux on sleeping asthmatics Am Rev Respir Dis 1990 141 6 1394 1399 10.1164/ajrccm/141.6.1394 2350084

[B13] Kjellen G Tibbling L Wranne B Bronchial obstruction after esophageal acid perfusion in asthmatics Clin Physiol 1981 1 3 285 292 10.1111/j.1475-097X.1981.tb00897.x 7199988

[B14] Singh V Aggarwal V Bansal S Nijhawan S Chaudhary N Effect of intraesophageal acid instillation on airway reactivity in patients with asthma J Assoc Physicians India 2000 48 6 601 602 11273538

[B15] Chakrabarti S Singh K Singh V Nain CK Jindal SK Airway response to acid instillation in esophagus in bronchial asthma Indian J Gastroenterol 1995 14 2 44 47 7797275

[B16] Herve P Denjean A Jian R Simonneau G Duroux P Intraesophageal perfusion of acid increases the bronchomotor response to methacholine and to isocapnic hyperventilation in asthmatic subjects Am Rev Respir Dis 1986 134 5 986 989 3096180 10.1164/arrd.1986.134.5.986

[B17] Vincent D Cohen-Jonathan AM Leport J Merrouche M Geronimi A Pradalier A Soule JC Gastro-esophageal reflux prevalence and relationship with bronchial reactivity in asthma Eur Respir J 1997 10 10 2255 2259 10.1183/09031936.97.10102255 9387949

[B18] Cuttitta G Cibella F Visconti A Scichilone N Bellia V Bonsignore G Spontaneous gastresophageal reflux and airway patency during the night in adult asthmatics Am J Respir Crit Care Med 2000 161 1 177 181 10.1164/ajrccm.161.1.9808014 10619817

[B19] Wu DN Tanifuji Y Kobayashi H Yamauchi K Kato C Suzuki K Inoue H Effects of esophageal acid perfusion on airway hyperresponsiveness in patients with bronchial asthma Chest 2000 118 6 1553 1556 10.1378/chest.118.6.1553 11115439

[B20] Wright RA Miller SA Corsello BF Acid-induced esophagobronchial-cardiac reflexes in humans Gastroenterology 1990 99 1 71 73 2344943 10.1016/0016-5085(90)91231-t

[B21] Lodi U Harding SM Coghlan HC Guzzo MR Walker LH Autonomic regulation in asthmatics with gastresophageal reflux Chest 1997 111 1 65 70 10.1378/chest.111.1.65 8995994

[B22] American Thoracic Society Lung function testing: selection of reference values and interpretative strategies Am Rev Respir Dis 1991 144 5 1202 1218 1952453 10.1164/ajrccm/144.5.1202

[B23] Demeester TR Johnson LF Joseph GJ Toscano MS Hall AW Skinner DB Patterns of gastresophageal reflux in health and disease Ann Surg 1976 184 4 459 470 10.1097/00000658-197610000-00009 13747 PMC1345443

[B24] Amarasiri DL Pathmeswaran A Dassanayake AS de Silva AP Ranasinha CD de Silva HJ Esophageal motility, vagal function and gastroesophageal reflux in a cohort of adult asthmatics BMC Gastroenterology 2012 12 140 10.1186/1471-230X-12-140 23057471 PMC3527260

[B25] Amarasiri LD Pathmeswaran A de Silva AP Dassanayake AS Ranasinha CD de Silva HJ Comparison of a composite symptom score assessing both symptom frequency and severity with a score that assesses frequency alone: a preliminary study to develop a practical symptom score to detect gastro-esophageal reflux disease in a resource-poor setting Eur J Gastroenterol Hepatol 2010 22 6 662 668 19491697 10.1097/MEG.0b013e32832cfe12

[B26] Ewing DJ Clarke BF Diagnosis and management of diabetic autonomic neuropathy Br Med J (Clin Res Ed) 1982 285 6346 916 918 10.1136/bmj.285.6346.916 PMC1500018 6811067

[B27] Wu DN Yamauchi K Kobayashi H Tanifuji Y Kato C Suzuki K Inoue H Effects of esophageal acid perfusion on cough responsiveness in patients with br onchial asthma Chest 2002 122 2 505 509 10.1378/chest.122.2.505 12171823

[B28] Shah PK Lakhotia M Mehta S Jain SK Gupta GL Clinical dysautonomia in patients with bronchial asthma Study with seven autonomic function tests. Chest 1990 98 6 1408 1413 10.1378/chest.98.6.1408 2245682

[B29] Gupta S Dolwani S A **study of autonomic status and its effect on ventilatory functions in bronchial asthma** Indian J Chest Dis Allied Sci 1996 38 3 147 156 8987288

[B30] Richter JE Delayed gastric emptying in reflux patients: to be or not to be? Am J Gastroenterol 1997 92 7 1077 1078 9219772

[B31] Araujo AC Aprile LR Dantas RO Terra-Filho J Vianna EO Bronchial responsiveness during esophageal acid infusion Lung 2008 186 2 123 128 10.1007/s00408-008-9072-z 18297339

[B32] Rosztóczy A Makk L Izbéki F Róka R Somfay A Wittmann T Asthma and gastroesophageal reflux: clinical evaluation of esophago-bronchial reflex and proximal reflux Digestion 2008 77 3–4 218 224 Epub 2008 Jul 19 18635940 10.1159/000146083

[B33] Andersen LI Schmidt A Bundgaard A Pulmonary function and acid application in the esophagus Chest 1986 90 3 358 363 10.1378/chest.90.3.358 3743147

[B34] Jiang SP Huang LW Role of gastresophageal reflux disease in asthmatic patients Eur Rev Med Pharmacol Sci 2005 9 3 151 160 16080634

[B35] Roka R Rosztoczy A Izbeki F Taybani Z Kiss I Lonovics J Wittmann T Prevalence of respiratory symptoms and diseases associated with gastroesophageal reflux disease Digestion 2005 71 92 96 10.1159/000084524 15775676

[B36] Davis RS Larsen GL Grunstein MM Respiratory response to intraesophageal acid infusion in asthmatic children during sleep J Allergy Clin Immunol 1983 72 4 393 398 10.1016/0091-6749(83)90505-5 6619453

[B37] Matthews PJ Knowles CH Chua YC Delaney C Hobson AR Aziz Q Effects of the concentration and frequency of acid infusion on the development and maintenance of esophageal hyperalgesia in a human volunteer model American Journal of Physiology Gastrointestinal and Liver Physiology 2008 294 4 G914 G917 10.1152/ajpgi.00445.2007 18258794

[B38] Cuomo R De Giorgi F Adinolfi L Sarnelli G Loffredo F Efficie E Verde C Savarese MF Usai P Budillon G Esophageal acid exposure and altered neurocardiac function in patients with GERD and idiopathic cardiac dysrhythmias Aliment Pharmacol Ther 2006 24 2 361 370 10.1111/j.1365-2036.2006.02987.x 16842463

